# Chemical Composition of *Scrophularia lucida* and the Effects on Tumor Invasiveness *in Vitro*

**DOI:** 10.3389/fphar.2018.00304

**Published:** 2018-04-03

**Authors:** Verena Lewenhofer, Lisa Schweighofer, Tobias Ledermüller, Julia Eichsteininger, Hanspeter Kählig, Martin Zehl, Chi H. Nguyen, Georg Krupitza, Ali Özmen, Liselotte Krenn

**Affiliations:** ^1^Department of Pharmacognosy, Faculty of Life Sciences, University of Vienna, Vienna, Austria; ^2^Clinical Institute of Pathology, Medical University of Vienna, Vienna, Austria; ^3^Department of Organic Chemistry, Faculty of Chemistry, University of Vienna, Vienna, Austria; ^4^Department of Analytical Chemistry, Faculty of Chemistry, University of Vienna, Vienna, Austria; ^5^Department of Clinical Pharmacy and Diagnostics, Faculty of Life Sciences, University of Vienna, Vienna, Austria; ^6^Department of Medicine I and Comprehensive Cancer Center, Medical University of Vienna, Vienna, Austria; ^7^Department of Biology, Faculty of Science and Art, Adnan Menderes University, Aydin, Turkey

**Keywords:** *Scrophularia lucida* L., iridoids, phenolic compounds, 2″-*O*-acetyl-homoplantaginin, hispidulin, intravasation, circular chemorepellent-induced defects (CCID) assay

## Abstract

A detannified methanolic extract of *Scrophularia lucida* L. attenuated the formation of cancer cell-induced circular chemorepellent induced defects (CCIDs) in the lymph endothelial cell barrier, which resemble entry ports for the intravasating tumor into the vasculature as a prerequisite for lymph node metastasis. Therefore, the composition of this extract was studied in an activity-guided approach. Since no data on the secondary metabolites of this plant were available, first phytochemical data were collected in the course of the fractionation of the extract. The study resulted in the identification of 14 substances, among them very rare iridoids, such as scrovalentinoside or koelzioside, and several flavonoids (e.g., nepitrin and homoplantaginin). One of the latter group, 2″-*O*-acetyl-homoplantaginin, is a new natural compound. In the most active fraction, the flavonoid hispidulin was identified as major component and the assay of the pure compound confirmed a contribution of hispidulin to the CCID-inhibitory effects of *S. lucida*. The activity of the two major iridoids in this assay was less compared to hispidulin.

## Introduction

Species of the genus *Scrophularia* have been applied for centuries in traditional medicine. The most important one among those, *Scrophularia ningpoensis* Hemsl., is widely used in traditional Chinese medicine for a broad spectrum of inflammatory diseases (De Santos Galíndez et al., 2002; Tong et al., 2006; Li et al., 2009) and listed in the Chinese Pharmacopeia against febrile diseases with excessive thirst or eruptions, constipation, cough due to exhaustion, conjunctivits, skin disorders, and others (Stöger, 2017). The use in the treatment of inflammatory disorders, wound healing, skin ailments, and health disturbances has been reported for numerous species such as *Scrophularia auriculata* L. or *Scrophularia canina* L. from the Mediterranean area (Giner et al., 2000; Guarrera and Lucia, 2007; Passalacqua et al., 2007) as well as for *Scrophularia variegata* M. Bieb. and *Scrophularia striata* Boiss. in traditional Iranian medicine (Azadmehr et al., 2013, 2015). A traditional use as an antipyretic and anti-inflammatory agent was described for *Scrophularia oldhamii* Oliv. and as a diuretic for *Scrophularia grossheimii* Schischkin (De Santos Galíndez et al., 2002). In Turkish traditional medicine, the species *Scrophularia nodosa* L. is well-known as a diuretic, to treat hemorrhoids, and also for wound healing, eruptive skin diseases, eczema, psoriasis, and pruritus (Baytop, 1999; Stevenson et al., 2002; Crisan et al., 2009). A use in these indications is also conveyed for the Turkish species *Scrophularia depauperata* Boiss., *Scrophularia cryptophila* Boiss. et. Heldr. Boiss., and *Scrophularia floribunda* Boiss. et. Bal. (Ozbilgin et al., 2014).

Recently, the activity of various extracts from several *Scrophularia* species has been studied in different cancer models: weak cytotoxic activity of an ethanolic extract from *S. striata* via induction of apoptosis and G_2_/M phase arrest was shown in Jurkat human leukemia cells (Azadmehr et al., 2013). In WEHI 164 fibrosarcoma cells, a methanolic extract of this plant exhibited a significant and dose-dependent reduction of the activity of matrix metalloproteinases (Hajiaghaee et al., 2007), which play a role in tissue remodeling and tumor invasion. An apolar fraction from a methanolic extract of *Scrophularia orientalis* L. significantly reduced the viability of neuroblastoma cells due to a loss of mitochondrial membrane integrity (Lange et al., 2016). In MCF-7 breast cancer cells, weak cytotoxicity of an ethanolic extract from *S. variegata* was reported (Azadmehr et al., 2015). A dichloromethane extract from *Scrophularia oxysepala* caused significant cytotoxic and apoptotic effects in MCF-7 cells by influencing the expression of p53, caspase 3, and c-myc, and the cleavage of PARP (Valiyari et al., 2013). The anti-tumor activity of *S. oxysepala* in breast cancer was confirmed *in vivo* in an allograft model in mice inoculated with mammary carcinoma 4T1 cells. Animals treated with 50 or 100 mg/kg of an ethanolic extract developed significantly smaller tumors in size and weight (Baradaran et al., 2017). In a study of five *Scrophularia* species, *S. floribunda* and *Scrophularia lucida* L. displayed the highest cytotoxic activity against HL-60 promyeloic leukemia cells. These extracts were also tested in a three-dimensional co-culture cell model consisting of MCF-7 breast cancer spheroids placed on top of telomerase-immortalized human lymph endothelial cell (LEC) monolayers. There, tumor spheroids cause the formation of large cell-free areas in the lymph endothelial barrier, so-called “circular chemorepellent induced defects” (CCIDs), through which tumor cells penetrate the vasculature. This assay mimics intravasation which is an early step within the metastatic cascade resembling the pathological situation in rodents and humans (Madlener et al., 2010; Vonach et al., 2011; Viola et al., 2013). A detannified methanolic extract of *S. lucida* L. was the most active one in suppressing CCID formation (Giessrigl et al., 2012).

The lack of any information about the chemical composition of *S. lucida* prompted us to perform an activity-guided search for the active principle of this species contributing to the inhibitory effect in the CCID assay.

## Experimental

### General Experimental Procedures

Hispidulin was obtained from Sigma–Aldrich. All other flavonoids for dereplication were on stock at the Department of Pharmacognosy, University of Vienna.

For TLC, silica gel plates or HPTLC plates RP-8 (Merck, Germany) and the mobile phases (1) CHCl_3_–CH_3_COOH_conc_.– MeOH–H_2_O (60:32:12:18), (2) EtOAc–HCOOH_conc._–CH_3_ COOH_conc_.–H_2_O (100:11:11:20), (3) EtOAc–MeOH–HCOOH_conc._–H_2_O (75:15:4:4), or (4) MeOH–H_2_O (90:10) were used.

A Shimadzu LC-20AD pump, an SPD M20A diode array detector, a CTO-20AC column oven, and a SIL 20AC HT auto-sampler (Kyoto, Japan) served for analytical HPLC on a Luna 5 μ C-18 (250 mm × 4.6 mm) column (Phenomenex, United States) at 25°C and a flow rate of 1 mL/min. Mobile phase 1 consisted of (A) aqueous formic acid (pH 3) and (B) acetonitrile-aqueous formic acid (pH 3; 80+2) and the gradient program 1 was 5% B (0 min), 20% B (15 min), 20% B (25 min), 50% B (65 min), and 95% B (80 min). Mobile phase 2 was (A) aqueous formic acid (pH 3) and (B) acetonitrile and the gradient 2 increased from 5% B (0 min) to 95% B (60 min). UV data were recorded from 190 to 400 nm. All solvents of analytical or HPLC-grade (acetonitrile, Chromanorm; methanol, LiChrosolv) were obtained from VWR International (West Chester, PA, United States) and CH_3_COOH_conc_. (Rotichrom) from Carl Roth (Karlsruhe, Germany).

NMR spectra were recorded on an *Avance* III 600 MHz NMR spectrometer (Bruker BioSpin, Germany) using a 5 mm broadband observe probe (BBFO Smart probe) with *z*-axis gradients and automatic tuning and matching accessory; resonance frequency for ^1^H NMR: 600.13 MHz, for ^13^C NMR: 150.90 MHz. The measurements were performed for solutions in fully deuterated DMSO or methanol at 298 K. Standard 1D and 2D experiments like double quantum filtered COSY, TOCSY, NOESY, HSQC, and HMBC were used as supplied by the manufacturer. Chemical shifts were referenced internally to the residual, non-deuterated solvent signal for ^1^H (δ = 2.50 ppm for DMSO, δ = 3.31 ppm for methanol) or to the carbon signal of the solvent for ^13^C (δ = 39.52 for DMSO, δ = 49.00 ppm for methanol). The analysis of the ^1^H NMR multiplett structures was assisted by spin simulations using DAISY within the Topspin Software (Bruker BioSpin).

LC–MS analyses were carried out on an UltiMate 3000 RSLC-series system (Dionex, Germering, Germany) coupled to a 3D quadrupole ion trap mass spectrometer via an orthogonal ESI source (HCT, Bruker Daltonics, Bremen, Germany). The HPLC parameters were as described above. The eluate flow was split 1:4 before entering the ESI ion source, which was operated as follows: capillary voltage: +3.5/-3.7 kV, nebulizer: 26 psi (N_2_), dry gas flow: 9 L/min (N_2_), and dry temperature: 340°C. Positive and negative ion mode multistage mass spectra up to MS^4^ were obtained in automated data-dependent acquisition mode using helium as collision gas, an isolation window of Δ*m/z* = 4, and a fragmentation amplitude of 1.0 V. In addition, compounds **13** and **14** were measured on the HCT instrument by direct infusion after addition of ammonium acetate as additive to generate [M+NH_4_]^+^ ions. HRESIMS spectra of compound **3** were recorded on a maXis HD ESI-Qq-TOF mass spectrometer (Bruker Daltonics) by direct infusion. The sum formulas of the detected ions were determined using Bruker Compass DataAnalysis 4.2 based on the mass accuracy (Δ*m*/*z* ≤ 2 ppm) and isotopic pattern matching (SmartFormula algorithm).

### Plant Material

Aerial parts of *S. lucida* were collected on April 20th 2012, along the Mediterranean coast east of Dalyan and south of Ortaca (latitude: 36.808838, longitude: 28.735583). The material was authenticated by Dr. Özkan Eren, Department of Biology, Adnan Menderes University, using the serial “Flora of Turkey and the East Aegean Islands” (Davis, 1968–1985). Voucher specimen (Herbarium code: AYDN No.: 2601) in duplicates was deposited in the herbarium of Department of Biology, Adnan Menderes University.

### Extraction and Isolation

The freeze-dried and pulverized material of *S. lucida* (1.8 kg) was extracted with methanol at the ratio of 1:10 at room temperature by maceration on a shaker overnight. The combined solutions were evaporated at 40°C resulting in 158 g crude methanol extract; 140 g of the extract were fractionated in four portions, whereby each portion was dissolved in 300 mL MeOH–H_2_O (10:1) and partitioned three times with 300 mL petroleum ether, each, to remove chlorophyll, waxes, and fatty matters. The MeOH–H_2_O phase was then diluted with 300 mL H_2_O and partitioned with three times 600 mL chloroform. After washing the organic layer with 1% aqueous NaCl solution and evaporation, 15.6 g chloroform fraction (CF) was obtained. From the remaining MeOH–H_2_O fraction, MeOH was removed and the aqueous residue partitioned with three times 280 mL ethyl acetate, resulting in 12.3 g ethyl acetate fraction (EF) and 110.2 g aqueous fraction (AF) after evaporation.

Ten grams of EF were separated by column chromatography (CC; 3 cm × 60 cm) on Sephadex LH-20 under elution with 50% MeOH yielding 15 combined fractions (a–o). In fractions k and l, compound **1** was identified by TLC and LC-DAD. Fractions e (1 g) and g (580 mg) were further fractionated on the same stationary phase under elution with 30% MeOH, resulting in fractions e1–e22 and g1–g19. From fraction e18, 46.5 mg of compound **2** precipitated. Solid phase extraction (Megabond Elut C18 cartridge; 12 mL; Agilent, Santa Clara, CA, United States; sequential elution with 20, 30, 40, and 50% MeOH) of fraction e22 resulted in the isolation of compound **3** (7 mg) and the identification of compound **4**. A major component from fraction g6 (63.2 mg) was finally purified by another CC on Sephadex LH-20 under elution with H_2_O to yield 27 mg compound **5**. In fraction g10, compounds **6** and **7** were identified, and in fraction g19, compound **8** was confirmed.

A portion of CF (3.9 g) was submitted to CC on silica and elution with CHCl_3_–MeOH–H_2_O (75:15:1.5), resulting in 25 fractions, five of which (A–E) were further investigated. In fraction A (62.5 mg), compound **9** was identified as the major component. In fraction B (150.8 mg), three major compounds (**10**–**12)** besides **8** were detected and tentatively identified by LC–MS. From fraction C (150.9 mg), compound **13** (144.7 mg) and from fraction D (630.5 mg), compound **14** (532 mg) were obtained. Fraction E (125.9 mg) contained a complex mixture of easy decomposable substances and attempts to isolate those by CC were not successful.

**Astragalin (1)**: Rf in TLC: system 1 = 0.50; system 2 = 0.74.

**Homoplantaginin (2)**: yellowish amorphous powder;^1^H-NMR and ^13^C-NMR data: see **Table 1**; +ESIMS *m/z* 463.1 [M+H]^+^; +ESIMS^2^ (463.1→) *m/z* 301.3 (100); +ESIMS^3^ (463.1 → 301.3 →) *m/z* 286.3 (100); +ESIMS^4^ (463.1 → 301.3 → 286.3 →) *m/z* 240.1 (10), 186.2 (28), 168.3 (100), 167.1 (11), 121.5 (25), and 120.5 (14); -ESIMS *m/z* 461.0 [M-H]^-^; -ESIMS^2^ (461.0 →) *m/z* 446.2 (45), 299.2 (100), 298.2 (17), 297.2 (16), 285.2 (20), 284.2 (73), and 283.2 (57); -ESIMS^3^ (461.0 → 299.2 →) *m/z* 284.2 (100); and -ESIMS^3^ (461.0 → 284.2 →) *m/z* 255.2 (100), 239.1 (10), 227.1 (51), 211.1 (21), 183.1 (11), and 137.2 (11).

**Table 1:** ^1^H NMR and ^13^C NMR data of compounds **2** and **3** in DMSO-d_6_.

		Compound 2			Compound 3		
Position		^1^H (ppm)	J_H.H_ (Hz)	^13^C (ppm)	^1^H (ppm)	J_H.H_ (Hz)	^13^C (ppm)
2	C	–	–	164.3	–	–	164.4
3	CH	6.86	s	102.7	6.87	s	102.7
4	C	–	–	182.3	–	–	182.3
4a	C	–	–	105.7	–	–	106.1
5	C	–	–	152.5	–	–	152.6
	OH	12.96	s	–	12.97	s	–
6	C	–	–	132.5	–	–	132.5
	CH_3_	3.77	s	60.3	3.65	s	60.2
7	C	–	–	156.5	–	–	155.9
8	CH	7.02		94.3	7.03	s	94.7
8a	C	–	–	152.1	–	–	152.0
1′	C	–	–	121.0	–	–	120.9
2′/6′	CH	7.95	d 8.8	128.6	7.94	d 8.9	128.6
3′/5′	CH	6.94	d 8.8	116.0	6.94	d 8.9	116.0
4′	C	–	–	161.4	–	–	161.5
1″	CH	5.11	d 7.1	100.2	5.35	d 8.1	97.9
2″	CH	3.34	dd 7.1/9.6	73.2	4.89	dd 8.1/9.6	73.3
	C	–	–		–	–	169.3
	CH_3_	–	–		2.04	s	20.8
3″	CH	3.32	dd 9.6/9.2	76.7	3.54	dd 9.6/9.1	73.8
4″	CH	3.21	dd 9.2/9.6	69.5	3.31	dd 9.1/9.7	69.6
5″	CH	3.47	ddd 9.6/ 6.0/2.1	77.3	3.58	ddd 9.7/6.0/2.1	77.5
6″a	CH_2_	3.73	dd 2.1/11.6	60.6	3.77	dd 2.1/11.8	60.4
b		3.49	dd 6.0/11.6		3.53	dd 6.0/11.8	
Further OH signals		10.40 5.43 5.12 4.63			10.43 5.39 4.77		

**2**″**-*O*-acetyl-homoplantaginin (3)**: yellowish amorphous powder;^1^H-NMR and ^13^C-NMR data: see **Table 1**; +ESIMS *m/z* 505.0 [M+H]^+^; +ESIMS^2^ (505.0 →) *m/z* 301.3 (100); +ESIMS^3^ (505.0 → 301.3 →) *m/z* 286.3 (100); +ESIMS^4^ (505.0 → 301.3 → 286.3 →) *m/z* 186.1 (18), 168.3 (100), 140.2 (20), and 121.4 (11); *-*ESIMS *m/z* 503.0 [M-H]^-^; -ESIMS^2^ (503.0 →) *m/z* 488.2 (9), 443.2 (10), 299.2 (100), 298.3 (20), 297.3 (20), 284.2 (45), and 283.2 (36); -ESIMS^3^ (503.0 → 299.2) *m/z* 284.2 (100); and -ESIMS^4^ (503.0 → 299.2 → 284.2 →) *m/z* 256.1 (59), 255.1 (100), 239.1 (24), 229.0 (28), 228.1 (20), 227.1 (23), 216.0 (27), 211.2 (23), 200.2 (24), 199.2 (38), 183.0 (12), 166.1 (17), 163.1 (13), 150.2 (13), and 141.2 (13). +HRESIMS *m/z* 505.1340 [M+H]^+^ (calcd for C_24_H_25_O_12_^+^, *m/z* 505.1341, Δ = 0.2 ppm), 527.1159 [M+Na]^+^ (calcd for C_24_H_24_O_12_Na^+^, *m/z* 527.1160, Δ = 0.2 ppm).

**Nepitrin (4)**: Rf in TLC: system 2 = 0.63. LC-DAD data: see Supplementary Material.

**Verbascoside (5)**: whitish amorphous powder; LC-DAD-data: see Supplementary Material; +ESIMS *m/z* 642.2 [M+NH_4_]^+^, 625.1 [M+H]^+^; +ESIMS^2^ (642.2 →) *m/z* 479.1 (18), 471.1 (74), and 325.0 (100); +ESIMS^3^ (642.2 → 471.1 →) *m/z* 453.2 (11), 325.0 (100), 309.0 (11), and 162.9 (54); +ESIMS^3^ (642.2 → 325.0 →) *m/z* 162.9 (100); +ESIMS^4^ (642.2 → 325.0 → 162.9 →) *m/z* 162.9 (16), 144.9 (100), and 134.9 (23); -ESIMS *m/z* 623.2 [M-H]^-^; -ESIMS^2^ (623.2 →) *m/z* 461.1 (100); and -ESIMS^3^ (623.2 → 461.1 →) *m/z* 315.0 (43), 160.8 (12), and 134.9 (100).

**Kaempferol-3-*O*-rutinoside (6)**: Rf in TLC: system 2 = 0.55. LC-DAD data: see Supplementary Material.

**Rutin (7)**: Rf in TLC: system 2 = 0.48. LC-DAD data: see Supplementary Material.

**Luteolin-7-*O*-glucoside (8)**: Rf in TLC: system 2 = 0.71. LC-DAD data: see Supplementary Material.

**Hispidulin (9):** yellowish amorphous powder; LC-DAD data: see Supplementary Material; +ESIMS *m/z* 301.1 [M+H]^+^; +ESIMS^2^ (301.1 →) *m/z* 286.0 (100); +ESIMS^3^ (301.1 → 286.0 →) *m/z* 256.8 (16), 185.9 (17), 167.9 (100), 140.0 (14), 122.0 (38), 120.9 (30), 119.0 (24), and 112.0 (23); -ESIMS *m/z* 299.0 [M-H]^-^; -ESIMS^2^ (299.0 →) *m/z* 283.9 (100); and -ESIMS^3^ (299.0 → 283.9 →) *m/z* 266.8 (17), 255.8 (56), 254.9 (29), 238.8 (22), 227.7 (64), 226.8 (74), 213.8 (43), 211.8 (55), 210.8 (18), 199.8 (77), 198.9 (10), 198.0 (10), 185.7 (32), 183.7 (28), 182.9 (17), 177.7 (11), 163.8 (24), 149.7 (21), 136.8 (100), and 129.8 (11).

**Buergeriside C1 isomer (10)**: +ESIMS *m/z* 307.1 [M-H_2_O+H]^+^, 347.1 [M+Na]^+^; +ESIMS^2^ (307.1 →) *m/z* 160.9 (100); +ESIMS^3^ (307.1 → 160.9 →) *m/z* 132.9 (100); -ESIMS *m/z* 359.2 [M+Cl]^-^, 369.1 [M+HCOO]^-^; and -ESIMS^2^ (369.1 →) *m/z* 323.0 (48), 262.9 (26), 218.8 (34), 190.8 (12), 176.8 (100), and 144.9 (27).

**Buergeriside C1 isomer (11):**+ESIMS *m/z* 307.1 [M-H_2_O+H]^+^, 347.1 [M+Na]^+^; +ESIMS^2^ (307.1 →) *m/z* 161.0 (100); +ESIMS^3^ (307.1 → 161.0 →) *m/z* 133.0 (100); -ESIMS *m/z* 359.3 [M+Cl]^-^, 369.1 [M+HCOO]^-^; and -ESIMS^2^ (369.1 →) *m/z* 323.9 (22), 323.0 (74), 236.8 (12), 219.7 (15), 218.8 (28), 190.8 (36), 176.9 (43), 145.9 (11), 144.8 (100), and 126.9 (7).

**6-O-(2**″**-Acetyl-3**″,**4**″**-*O*-di-*trans*-cinnamoyl-α-L-rhamnopyranosyl-catalpol (12)**: +ESIMS *m/z* 833.3 [M+Na]^+^; +ESIMS^2^ (833.3 →) *m/z* 803.2 (11), 773.2 (35), 751.3 (100), 495.1 (30); -ESIMS *m/z* 845.5 [M+Cl]^-^, 855.3 [M+HCOO]^-^; -ESIMS^2^ (855.3 →) *m/z* 810.2 (65), 809.3 (100), and 533.2 (14); and -ESIMS^3^ (855.3 → 809.3 →) *m/z* 767.2 (36), 750.2 (28), 749.2 (37), 662.1 (39), 661.2 (100), 648.1 (41), 647.2 (36), 629.2 (11), 620.2 (16), 619.2 (21), 618.3 (12), 617.0 (17), 533.1 (16), 353.0 (11), 311.0 (18), and 293.0 (11).

**Koelzioside (13)**: whitish amorphous powder;^1^H-NMR and ^13^C-NMR shifts: see Supplementary Material; +ESIMS *m/z* 828.4 [M+NH_4_]^+^; +ESIMS^2^ (828.4 →) *m/z* 449.1 (100); +ESIMS^3^ (828.4 → 449.1 →) *m/z* 389.0 (11), 301.0 (47), 241.0 (25), and 131.0 (100); and -ESIMS *m/z* 845.3 [M+Cl]^-^, 855.3 [M+HCOO]^-^.

**Scrovalentinoside (14)**: whitish amorphous powder;^1^H-NMR and ^13^C-NMR shifts: see Supplementary Material; +ESIMS *m/z* 770.3 [M+NH_4_]^+^; +ESIMS^2^ (770.3 →) *m/z* 770.3 (14), 591.2 (5), 573.1 (4), and 391.1 (100); +ESIMS^3^ (770.3 → 391.1 →) *m/z* 391.1 (28), 271.1 (6), 213.0 (21), 161.0 (100), 153.0 (71), 133.0 (7), and 111.1 (19); and -ESIMS *m/z* 787.3 [M+Cl]^-^, 797.3 [M+HCOO]^-^.

### Cell Culture

Cultivation of human MCF-7 breast cancer cells was carried out in MEM medium supplemented with 10% FCS, 1% penicillin/streptomycin, and 1% NEAA (all from Invitrogen Life Technologies, Karlsruhe, Germany) and of telomerase immortalized human LECs in EGM2 MV medium (Clonetics CC-4147, Allendale, NJ, United States) at 37°C in a humidified atmosphere containing 5% CO_2_.

### 3-D Co-cultivation of MCF-7 Cancer Cells With LECs

MCF-7 cells were transferred to 30 mL MEM medium containing 6 mL of a 1.6% methylcellulose solution (0.3% final concentration; cat. no. M-512, 4000 centipoises; Sigma–Aldrich, Munich, Germany). To each well of a 96-well plate (Greiner Bio-one, Cellstar 650185, Kremsmünster, Austria), 150 μL of the cell suspension was transferred for spheroid formation within 48 h. Then, MCF-7 spheroids were washed in PBS and transferred to CellTracker^TM^ green (Invitrogen Life Technologies, Karlsruhe, Germany) stained LEC monolayers that had been seeded into 24-well plates (Costar 3524, Sigma) in 2 mL EGM2 MV medium (Vonach et al., 2011).

### Circular Chemorepellent-Induced Defect (CCID) Assay

After 4 h of incubation of the co-cultures of MCF-7 spheroids (3,000 cells/spheroid) and the LEC monolayer, the size of CCIDs in the LEC monolayer underneath the MCF-7 spheroids was photographed with an Axiovert (Zeiss, Jena, Germany) fluorescence microscope to visualize CellTracker^TM^ (green) stained LECs underneath the spheroids (Madlener et al., 2010). CCID areas were calculated with the Axiovision Re. 4.5 software (Zeiss, Jena, Germany). MCF-7 spheroids were treated with solvent (ethanol) as negative control. The CCID areas of at least 15 spheroids per experiment were measured.

## Results and Discussion

**FIGURE 1:**
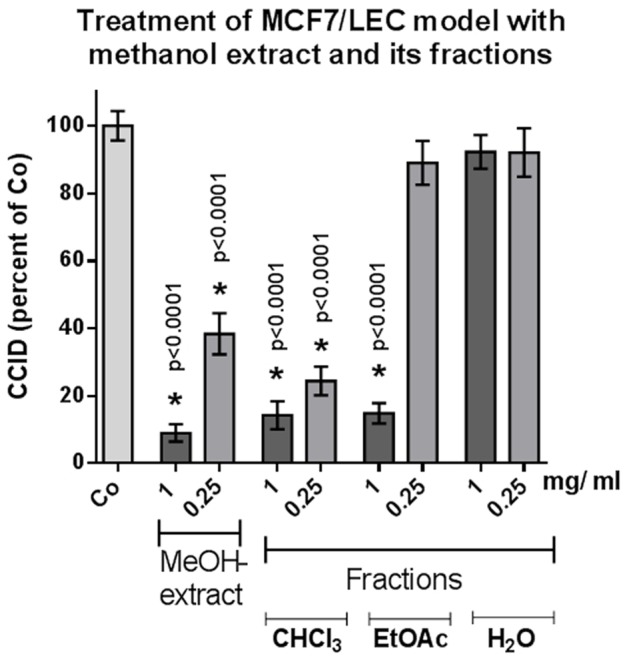
MCF-7 spheroids pre-treated for 30 min with solvent (Co) or indicated concentration of different fractions, placed on top of LEC monolayers, and co-cultivated for 4 h when CCIDs were analyzed. Three independent experiments with at least 15 replicates were analyzed. Error bars indicate means ± SEM and asterisks significance (*t*-test).

**FIGURE 2:**
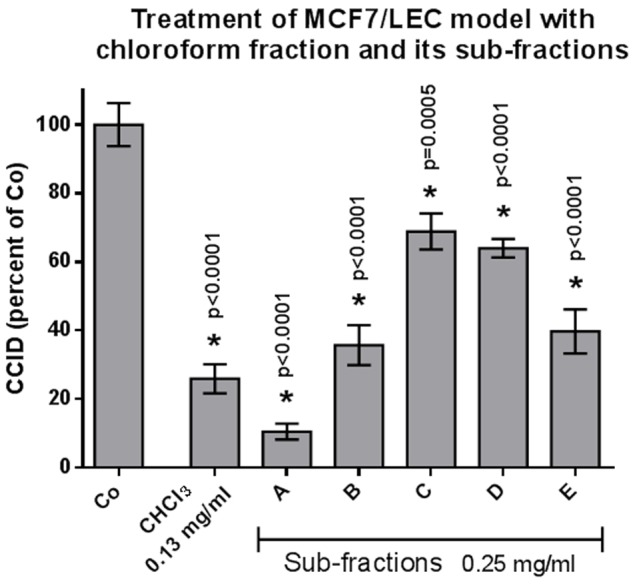
MCF-7 spheroids pre-treated for 30 min with solvent (Co) or indicated concentration of different subfractions, placed on top of LEC monolayers, and co-cultivated for 4 h when CCIDs were analyzed. Three independent experiments with at least 15 replicates were analyzed. Error bars indicate means ± SEM and asterisks significance (*t*-test).

From the methanolic extract of the aerial parts from *S. lucida*, apolar compounds were depleted by extraction with petroleum ether. After subsequent partition with chloroform and ethyl acetate, the two organic fractions and the residue of the aqueous phase were tested in the CCID assay. The crude methanol extract significantly and linearly inhibited CCID formation between concentrations of 0.25–1 mg/mL (**Figure 1**). At a concentration of 0.25 mg/mL, the CF seemed to be even more effective than 0.25 mg/mL of the methanol extract; however, this difference was not significant. The EF was only active at 1 mg/mL and the AF was inactive at the tested concentrations. Therefore, the CF was chosen for further separation by CC on silica and five of the resulting sub-fractions (A–E) were active in the CCID assay with sub-fraction A exhibiting the strongest inhibition (**Figure 2**). The EF was separated by size exclusion chromatography followed by solid phase extraction or another subsequent size exclusion chromatography. Due to the lower activity of the EF in the CCID assay, these sub-fractions were submitted to chemical investigations only.

**FIGURE 3:**
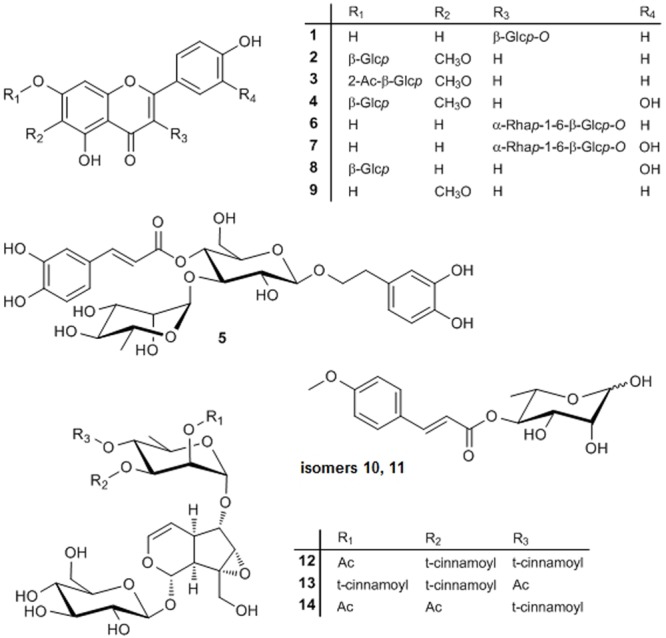
Structures of compounds **1**–**14**. Glcp, glucopyranosyl; Rhap, rhamnopyranosyl; Ac, acetyl.

The processes resulted in the elucidation of 14 compounds, one of which is a new natural product (**Figure 3**). The 13 known compounds were identified by chromatographic, mass spectrometric and/or spectroscopic dereplication with authentic samples or data from literature. This led to the elucidation of seven flavonoids. The widespread natural compounds astragalin (**1**), luteolin-7-*O*-glucoside (**8**), kaempferol-3-*O*-rutinoside (**6**), and rutin (**7**) were confirmed by TLC- and LC-DAD comparison with the authentic substances. To the best of our knowledge, only the two latter have been reported in the genus *Scrophularia* until now, namely, in *Scrophularia ilwensis* C. Koch (Calis et al., 1993). Two further, less prevalent flavonoids, nepitrin (**4**) and homoplantaginin (**2**), had been isolated from *S. ningpoensis* (Li et al., 2009) and nepitrin also from *S. striata* (Monsef-Esfahani et al., 2010) before. ^1^H-NMR- and ^13^C-NMR data of **2** correlated excellently with the data published earlier for homoplantaginin (Wang et al., 1998; Dawa et al., 2009). Hispidulin (**9**) was detected for the first time in a *Scrophularia* species and elucidated by LC-DAD comparison and LC–MS data.

A new flavonoid (**3**) was identified by thorough MS and NMR experiments: a molecular formula of C_24_H_24_O_12_ was determined from the HRESIMS data. Fragmentation of the [M+H]^+^ ion at *m/z* 505.0 showed the loss of a 204 Da to yield a Y_0_ ion at *m/z* 301.3, which again lost a ${\mathrm{CH}}_{3}^{∙}$ in MS^3^, typical for a methoxy group bound to an aromatic system. The MS^2^, MS^3^, and MS^4^ spectra were nearly identical to those of homoplantaginin. Fragmentation of the [M-H]^-^ ion at *m/z* 503.0 confirmed this information. In addition, the loss of 60 Da (CH_3_COOH) in MS^2^ indicated acetylation of the glucose of homoplantaginin. The signals of **3** in the ^1^H-NMR spectrum excellently correlated with those of compound **2** (**Table 1**), despite a large downfield shift of the proton at C-2″ in the sugar for 1.55 ppm. The signals of the protons at C-1″ and C-3″ were shifted downfield for 0.24 and 0.22 ppm, respectively, as well. Additionally, from the singlet at δ = 2.038 ppm, linkage of an acetyl group was deduced. The connection of an additional acetyl group in position 2″ of homoplantaginin was confirmed by the ^13^C-NMR data. The signals of C-1″ and C-3″ were shifted upfield for 2.28 and 2.95 ppm, respectively. Signals of an acetyl group occurred at δ = 169.26 ppm (quarternary C) and δ = 20.82 ppm (methyl C). An HMBC crosspeak from the CH_3_ protons to the C-2″ finally proofed the connectivity. Thus, compound **3** was unambiguously identified as 2″-*O*-acetyl-homoplantaginin.

The phenylpropanoid verbascoside, which had been detected in many species of the Scrophulariaceae family (see, e.g., Zhang et al., 2013; Pasdaran et al., 2016, 2017; Venditti et al., 2016), was isolated from *S. lucida* as well and identified by HPLC-DAD and MS.

The genus is well-known for synthesizing a broad range of iridoids (De Santos Galíndez et al., 2002) and two such compounds were isolated from and two further ones tentatively identified in the CF.

The major iridoid was scrovalentinoside (**14**). The compound was elucidated by MS and NMR experiments (see Supplementary Material). The spectroscopic data were in excellent accordance with those published after isolation of the compound from *S. auriculata* L. ssp. *pseudoauriculata* (Senn.) Bolós et Vigo (Giner et al., 1998) and from *S. nodosa* L. (Miyase and Mimatsu, 1999). A second iridoid was unambiguously elucidated as koelzioside (**13**) based on MS experiments and the analogy of the ^1^H-NMR and ^13^C-NMR data as reported for the compound, until now only known from *Scrophularia koelzii* L. (Bhandari et al., 1992) and *Scrophularia deserti* Del. (Ahmed et al., 2003). Tentatively identified by thorough LC–MS experiments were two isomers of buergeriside C_1_ (**10** and **11**), known from *Scrophularia buergeriana* (Miquel) (Kim and Kim, 2000), and 6-*O*-(2″-acetyl-3″,4″-di-*O-trans*-cinnamoyl)-α-L-rhamnopyranosyl-catalpol (scropolioside B, **12**), reported in *Scrophularia scopolii* [Hoppe ex] Pers. var. *scopolii* (Calis et al., 1988). Compounds **10** and **11** both showed a UV spectrum typical for a para-substituted cinnamic acid derivative and yielded identical fragment ions, although the relative intensities were different in the MS^2^ spectra of the [M+COOH]^-^ ion of **10** and **11**. Fragmentation of the [M-H_2_O+H]^+^ ion yielded the acylium ion of methoxycinnamic acid (Z_0_), while CID of the [M+COOH]^-^ ion produced deprotonated methoxycinnamic acid (Y_0_), dehydrated deoxyhexose (B_1_), and typical cross-ring cleavage of the deoxyhexose. From the compounds described for the genus *Scrophularia*, this pattern only fits to buergeriside C_1_, why we have tentatively identified **10** and **11** as buergeriside C_1_ isomers. The UV spectrum of **12** points toward cinnamic acid. Fragmentation of the [M-H]^-^ ion (resulting from CID of the [M+COOH]^-^ ion) yielded the neutral loss of 60, 148, and 162 Da, fitting to an acetylated, cinnamoylated, and hexosylated compound. Scropolioside B is the only previously described compound in the genus *Scrophularia* being in accordance with these data. All iridoids detected in *S. lucida* are very rare natural compounds.

The quantitative determination by HPLC of the major compounds in the methanolic extract revealed 3% homoplantaginin, 1.4% scrovalentinoside, and 0.7% koelzioside.

In an earlier study, 20 μg/mL of the methanolic extract of *S. lucida* was reported to induce 70% apoptosis and to almost completely inhibit proliferation of HL-60 cells in S-phase within 48 and 72 h, respectively (Giessrigl et al., 2012). Here, we demonstrate that 250 μg/mL of the methanolic extract inhibits 60% CCID formation in the MCF-7/LEC model within 4 h and the CF of the methanolic extract inhibited CCIDs almost similarly (with insignificant difference; **Figure 1**). Sub-fraction A of the CF was the most active one and other four fractions (B–E) also exhibited significant CCID-inhibitory properties (**Figure 2**).

Hispidulin turned out to be the major component in sub-fraction A, whereas koelzioside and scrovalentinoside, the main components in sub-fractions C and D, respectively, showed significantly less activity. To evaluate the potential of hispidulin in suppressing CCID formation, the pure compound was tested as well-demonstrating an IC_50_ of 88.7 μM confirming a contribution of hispidulin to the effects of the methanolic extract. This further implicated that other components present in sub-fraction A must have synergized strongly with hispidulin as only 250 μg/mL of sub-fraction A inhibited ∼90% CCID formation (compared to 26.6 mg/mL of purified hispidulin, which inhibited just 50%). Conceivably, residual amounts of sub-fraction B still been present in sub-fraction A caused this synergy. Hispidulin, *in vitro* and *in vivo*, had been shown to inhibit proliferation and to induce apoptosis of various cancer lines (Yu et al., 2013; Gao et al., 2015; Wang et al., 2015). The compound exhibited cytotoxicity toward HepG2 liver cancer cells but not normal liver cells (Gao et al., 2014). It also inhibited pancreas cancer cell mobility and invasion *in vitro* as well as neovascularization in C57/BL6 mice (He et al., 2011). Additionally, colon cancer cell intravasation through blood endothelial barriers was hindered *in vitro* (Holzner et al., 2017).

## Conclusion

In the activity-guided search for components of *S. lucida* inhibitory in the CCID assay, we showed that intravasation of breast cancer cells through the lymph endothelial wall, which is the major route of breast cancer dissemination, is significantly inhibited by hispidulin and other compounds present in the CF of *S. lucida* methanolic extract.

In addition, this is the first study characterizing the chemical composition of *S. lucida*. It resulted in the proof or tentative identification of several rare iridoids and flavonoids for the first time in the genus *Scrophularia* and a new compound, 2″-*O*-acetyl-homoplantaginin, was unambiguously elucidated.

For a more profound assessment of the potential of *S. lucida* to inhibit metastasis in breast cancer, testing in an appropriate animal model is necessary as a next step.

## Author Contributions

VL, LS, and TL performed the phytochemical fractionation of the extract and isolation of secondary metabolites. JE performed the CCID assay of hispidulin. HK performed all NMR measurements and structure elucidation. MZ performed all MS and LC–MS measurements and structure elucidation. CN performed the CCID assays of the extracts and fractions. GK supervised all pharmacological experiments. AÖ collected the plant material, was responsible for correct identification, and prepared the extract. LK supervised all phytochemical work. LK, HK, MZ, CN, and GK compiled the manuscript.

## Conflict of Interest Statement

The authors declare that the research was conducted in the absence of any commercial or financial relationships that could be construed as a potential conflict of interest.

## References

[B1] Ahmed B. Al-Rehaily A. J. Al-Howiriny T. A. El-Sayed K. A. Ahmad M. S. (2003). Scropolioside-D2 and harpagoside-B: two new iridoid glycosides from *Scrophularia deserti* and their antidiabetic and antiinflammatory activity. *Biol. Pharm. Bull.* 26 462–467. 10.1248/bpb.26.462 12673026

[B2] Azadmehr A. Hajiaghaee R. Baradaran B. Haghdoost-Yazdi H. (2015). Apoptosis cell death effect of *Scrophularia variegata* on breast cancer cells via mitochondrial intrinsic pathway. *Adv. Pharm. Bull.* 5 443–446. 10.15171/apb.2015.060 26504768 PMC4616888

[B3] Azadmehr A. Hajiaghaee R. Mazandarani M. (2013). Induction of apoptosis and G2 /M cell cycle arrest by *Scrophularia striata* in a human leukaemia cell line. *Cell Prolif.* 46 637–643. 10.1111/cpr.12074 24460717 PMC6496267

[B4] Baradaran P. C. Mohammadi A. Mansoori B. Baradaran S. C. Baradaran B. (2017). Growth inhibitory effect of *Scrophularia oxysepala* extract on mouse mammary carcinoma 4T1 cells in vitro and in vivo systems. *Biomed. Pharmacother.* 85 718–724. 10.1016/j.biopha.2016.11.086 27923691

[B5] Baytop T. (1999). *Türkiyede Bitkiler ile Tedavi.* Istanbul: Istanbul University Press.

[B6] Bhandari S. P. S. Mishra A. Roy R. Garg H. S. (1992). Koelzioside, an iridoid diglycoside from *Scrophularia koelzii*. *Phytochemistry* 31 689–691. 10.1016/0031-9422(92)90061-T

[B7] Calis I. Gross G. A. Winkler T. Sticher O. (1988). Isolation and structure elucidation of two highly acylated iridoid diglycosides from *Scrophularia scopolii*. *Planta Med.* 54 168–170. 10.1055/s-2006-962382 17265233

[B8] Calis I. Zor M. Basaran A. A. Wright A. D. Sticher O. (1993). Karsoside and scropolioside D, two new iridoid glycosides from *Scrophularia ilwensis*. *J. Nat. Prod.* 56 606–609. 10.1021/np50094a022 8496707

[B9] Crisan G. Kiss B. Vlase L. Balica G. Tamas M. (2009). HPLC determination of some phenolic compounds of *Scrophularia nodosa* and *S. scopolii*. *Chem. Nat. Compd.* 45 885–888. 10.1007/s10600-010-9478-8

[B10] Davis P. (1968–1985). *Flora of Turkey.* Edinburgh: Edinburgh University Press 1–10.

[B11] Dawa Z. Bai Y. Zhou Y. Gesang S. Ping A. Ding L. (2009). Chemical constituents of the whole plants of *Saussurea medusa*. *J. Nat. Med.* 63 327–330. 10.1007/s11418-009-0320-1 19219524

[B12] De Santos Galíndez J. Díaz Lanza A. M. Matellano L. F. (2002). Biologically active substances from the genus *Scrophularia*. *Pharm. Biol.* 40 45–59. 10.1076/phbi.40.1.45.5864

[B13] Gao H. Jiang Q. Han Y. Peng J. Wang C. (2015). Hispidulin potentiates the antitumor effect of sunitinib against human renal cell carcinoma in laboratory models. *Cell Biochem. Biophys.* 71 757–764. 10.1007/s12013-014-0260-6 25260394

[B14] Gao H. Wang H. Peng J. (2014). Hispidulin induces apoptosis through mitochondrial dysfunction and inhibition of P13k/Akt signalling pathway in HepG2 cancer cells. *Cell Biochem. Biophys.* 69 27–34. 10.1007/s12013-013-9762-x 24068521

[B15] Giessrigl B. Yazici G. Teichmann M. Kopf S. Ghassemi S. Atanasov A. G. (2012). Effects of *Scrophularia* extracts on tumor cell proliferation, death and intravasation through lymphoendothelial cell barriers. *Int. J. Oncol.* 40 2063–2074. 10.3892/ijo.2012.1388 22367166

[B16] Giner R. M. Villalba M. L. Del Carmen Recio M. Mainez S. Gray A. I. Rios J. L. (1998). A New Iridoid from *Scrophularia auriculata* ssp. pseudoauriculata. *J. Nat. Prod.* 61 1162–1163. 10.1021/np980067o 9748391

[B17] Giner R. M. Villalba M. L. Recio M. C. Máñez S. Cerdá-Nicolás M. Ríos J. L. (2000). Anti-inflammatory glycoterpenoids from *Scrophularia auriculata*. *Eur. J. Pharmacol.* 389 243–252. 10.1016/S0014-2999(99)00846-8 10688990

[B18] Guarrera P. M. Lucia L. M. (2007). Ethnobotanical remarks on central and southern Italy. *J. Ethnobiol. Ethnomed.* 3:23. 10.1186/1746-4269-3-23 17537240 PMC1906747

[B19] Hajiaghaee R. Monsef-Esfahani H. R. Khorramizadeh M. R. Saadat F. Shahverdi A. R. Attar F. (2007). Inhibitory effect of aerial parts of *Scrophularia striata* on matrix metalloproteinases expression. *Phytother. Res.* 21 1127–1129. 10.1002/ptr.2221 17639554

[B20] He L. Wu Y. Lin L. Wang J. Wu Y. Chen Y. (2011). Hispidulin, a small flavonoid molecule, suppresses the angiogenesis and growth of human pancreatic cancer by targeting vascular endothelial growth factor receptor 2-mediated PI3K/Akt/mTOR signaling pathway. *Cancer Sci.* 102 219–225. 10.1111/j.1349-7006.2010.01778.x 21087351

[B21] Holzner S. Brenner S. Atanasov A. G. Senfter D. Stadler S. Nguyen C. H. (2017). Intravasation of SW620 colon cancer cell spheroids through the blood endothelial barrier is inhibited by clinical drugs and flavonoids in vitro. *Food Chem. Toxicol.* 111 114–124. 10.1016/j.fct.2017.11.015 29129665

[B22] Kim S. R. Kim Y. C. (2000). Neuroprotective phenylpropanoid esters of rhamnose isolated from roots of Scrophularia buergeriana. *Phytochemistry* 54 503–509. 10.1016/S0031-9422(00)00110-2 10939354

[B23] Lange I. Moschny J. Tamanyan K. Khutsishvili M. Atha D. Borris R. P. (2016). *Scrophularia orientalis* extract induces calcium signaling and apoptosis in neuroblastoma cells. *Int. J. Oncol.* 48 1608–1616. 10.3892/ijo.2016.3373 26848085 PMC4777595

[B24] Li J. Huang X. Du X. Sun W. Zhang Y. (2009). Study of chemical composition and antimicrobial activity of leaves and roots of Scrophularia ningpoensis. *Nat. Prod. Res.* 23 775–780. 10.1080/14786410802696247 19418360

[B25] Madlener S. Saiko P. Vonach C. Viola K. Huttary N. Stark N. (2010). Multifactorial anticancer effects of digalloyl-resveratrol encompass apoptosis, cell-cycle arrest, and inhibition of lymphendothelial gap formation in vitro. *Br. J. Cancer* 102 1361–1370. 10.1038/sj.bjc.6605656 20424615 PMC2865764

[B26] Miyase T. Mimatsu A. (1999). Acylated iridoid and phenylethanoid glycosides from the aerial parts of *Scrophularia nodosa*. *J. Nat. Prod.* 62 1079–1084. 10.1021/np9805746 10479307

[B27] Monsef-Esfahani H. R. Hajiaghaee R. Shahverdi A. R. Khorramizadeh M. R. Amini M. (2010). Flavonoids, cinnamic acid and phenyl propanoid from aerial parts of *Scrophularia striata*. *Pharm. Biol.* 48 333–336. 10.3109/13880200903133829 20645822

[B28] Ozbilgin A. Durmuskahya C. Kayalar H. Ostan I. (2014). Assessment of in vivo antimalarial activities of some selected medicinal plants from Turkey. *Parasitol. Res.* 113 165–173. 10.1007/s00436-013-3639-1 24146207

[B29] Pasdaran A. Delazar A. Ayatollahi S. A. Nahar L. Sarker S. D. (2016). Phytochemical and bioactivity evaluation of *Scrophularia amplexicaulis* Benth. *Rec. Nat. Prod.* 10 519–525.

[B30] Pasdaran A. Pasdaran A. Delazar A. Ayatollahi S. A. Ayatollahi S. A. Pasdaran A. (2017). Chemical composition and biological activities of methanolic extract of *Scrophularia oxysepala* Boiss. *Iran. J. Pharm. Res.* 16 338–346. 28496487 PMC5423259

[B31] Passalacqua N. G. Guarrera P. M. De Fine G. (2007). Contribution to the knowledge of the folk plant medicine in Calabria region (Southern Italy). *Fitoterapia* 78 52–68. 10.1016/j.fitote.2006.07.005 17084993

[B32] Stevenson P. C. Simmonds M. S. J. Sampson J. Houghton P. J. Grice P. (2002). Wound healing activity of acylated iridoid glycosides from *Scrophularia nodosa*. *Phytother. Res.* 16 33–35. 10.1002/ptr.798 11807962

[B33] Stöger E. A. (2017). *Arzneibuch der Chinesischen Medizin.* Stuttgart: Deutscher Apotheker Verlag.

[B34] Tong S. Yan J. Lou J. (2006). Preparative isolation and purification of harpagoside from *Scrophularia ningpoensis* Hemsley by high-speed counter-current chromatography. *Phytochem. Anal.* 17 406–408. 10.1002/pca.938 17144248

[B35] Valiyari S. Jahanban-Esfahlan R. Shahneh F. Z. Yaripour S. Baradaran B. Delazar A. (2013). Cytotoxic and apoptotic activity of *Scrophularia oxysepala* in MCF-7 human breast cancer cells. *Toxicol. Environ. Chem.* 95 1208–1220. 10.1080/02772248.2013.854362

[B36] Venditti A. Frezza C. Riccardelli M. Foddai S. Nicoletti M. Serafini M. (2016). Secondary metabolites from *Scrophularia canina* L. *Nat. Prod. Res.* 30 1665–1669. 10.1080/14786419.2015.1122598 26675659

[B37] Viola K. Kopf S. Huttary N. Vonach C. Kretschy N. Teichmann M. (2013). Bay11-7082 inhibits the disintegration of the lymphendothelial barrier triggered by MCF-7 breast cancer spheroids; the role of ICAM-1 and adhesion. *Br. J. Cancer* 108 564–569. 10.1038/bjc.2012.485 23093227 PMC3593529

[B38] Vonach C. Viola K. Giessrigl B. Huttary N. Raab I. Kalt R. (2011). NF-kappaB mediates the 12(S)-HETE-induced endothelial to mesenchymal transition of lymphendothelial cells during the intravasation of breast carcinoma cells. *Br. J. Cancer* 105 263–271. 10.1038/bjc.2011.194 21629247 PMC3142797

[B39] Wang M. Li J. Rangarajan M. Shao Y. Lavoie E. J. Huang T.-C. (1998). Antioxidative phenolic compounds from sage (Salvia officinalis). *J. Agric. Food Chem.* 46 4869–4873. 10.1021/jf980614b

[B40] Wang Y. Liu W. He X. Fei Z. (2015). Hispidulin enhances the anti-tumor effects of temozolomide in glioblastoma by activating AMPK. *Cell Biochem. Biophys.* 71 701–706. 10.1007/s12013-014-0252-6 25315637

[B41] Yu C. Y. Su K. Y. Lee P. L. Jhan J. Y. Tsao P. H. Chan D. C. (2013). Potential therapeutic role of hispidulin in gastric cancer through induction of apoptosis via NAG-1 signaling. *Evid. Based Complement. Alternat. Med.* 2013:518301. 10.1155/2013/518301 24159347 PMC3789485

[B42] Zhang L. Yang Z. Jia Q. Dorje G. Zhao Z. Guo F. (2013). Two new phenylpropanoid glycosides with interesterification from *Scrophularia dentata* Royle ex Benth. *J. Mol. Struct.* 1049 299–302. 10.1016/j.molstruc.2013.05.039

