# Supplementary material for: Chemical Composition of Scrophularia lucida and the Effects on Tumor Invasiveness in Vitro
Source: Front Pharmacol. 2018 Apr 3;9:304. doi: 10.3389/fphar.2018.00304 (PMC5891616; doi:10.3389/fphar.2018.00304)
Supplement: Supplementary file 1 [file Data_Sheet_1.DOCX]

**Supporting Information**

**Chemical composition of *Scrophularia lucida* and the effects on tumor invasiveness *in vitro***

Verena Lewenhofer^1^, Lisa Schweighofer^1^, Tobias Ledermüller^1^, Julia Eichsteininger^1,5^, Hanspeter Kählig^2^, Martin Zehl^1,3^, Chi Huu Nguyen^4,5,6^, Georg Krupitza^5^, Ali Özmen^7*^, Liselotte Krenn^1*^

^1^ Department of Pharmacognosy, Faculty of Life Sciences, University of Vienna, Vienna, Austria

^2^ Department of Organic Chemistry, Faculty of Chemistry, University of Vienna, Vienna, Austria

^3^ Department of Analytical Chemistry, Faculty of Chemistry, University of Vienna, Vienna, Austria

^4^ Department of Clinical Pharmacy and Diagnostics, Faculty of Life Sciences, University of Vienna, Vienna, Austria

^5^ Clinical Institute of Pathology, Medical University of Vienna, Vienna, Austria

^6^ Department of Medicine I and Comprehensive Cancer Center, Medical University of Vienna, Vienna, Austria

^7^ Department of Biology, Faculty of Science and Art, Adnan Menderes University, Aydin, Turkey

* Equal Corresponding authors:

Liselotte Krenn

liselotte.krenn@univie.ac.at

Ali Özmen

[aozmen@adu.edu.tr](mailto:aozmen@adu.edu.tr)

Figure 1: Structures of koelzioside (**13**) and scrovalentinoside (**14**)

Table 1: ^1^H NMR and ^13^C NMR data of koelzioside (**13**) in MeOH-d_4_; 298 K

| **Position** | | **^1^H (ppm)** | **J_H.H_ (Hz)** | **^13^C (ppm)** | **^13^C (ppm)** (Bhandari et al., 1992) |
| --- | --- | --- | --- | --- | --- |
| 1 | CH | 5.116 | d 9.7 | 95.13 | 95.11 |
| 3 | CH | 6.409 | d 5.9 / d 1.8 | 142.50 | 142.47 |
| 4 | CH | 5.108 | d 5.9 / d 4.7 | 103.18 | 103.18 |
| 5 | CH | 2.522 | d 7.6 / d 7.9 / d 4.5 / d 1.8 | 37.17 | 37.13 |
| 6 | CH | 4.105 | d 8.2 / d 1.0 | 84.92 | 84.91 |
| 7 | CH | 3.707 | d 1.0 | 59.45 | 59.43 |
| 8 | C | --- | --- | 66.55 | 66.53 |
| 9 | CH | 2.606 | d 7.6 / d 9.7 | 43.28 | 43.23 |
| 10 | CH_2_ | 4.168 | d 13.2 | 61.43 | 61.40 |
|  |  | 3.828 | d 13.2 |  |  |
| β-Glc |  |  |  |  |  |
| 1 | CH | 4.786 | d 7.9 | 99.71 | 99.68 |
| 2 | CH | 3.273 | d 7.9 / d 9.2 | 74.82 | 74.77 |
| 3 | CH | 3.406 | d 9.2 / d 8.8 | 77.67 | 78.58 |
| 4 | CH | 3.260 | d 8.8 / d 9.8 | 71.78 | 71.71 |
| 5 | CH | 3.323 | d 9.8 / d 2.1 / d 6.7 | 78.65 | 77.61 |
| 6 | CH_2_ | 3.920 | d 2.1 / d 11.9 | 62.96 | 62.91 |
|  |  | 3.631 | d 6.7 / d 11.9 |  |  |
| α-Rha |  |  |  |  |  |
| 1 | CH | 5.173 | d 1.8 | 97.80 | 97.77 |
| 2 | CH | 5.501 | d 1.8 / d 3.5 | 71.41 | 71.38 |
| 3 | CH | 5.454 | d 3.5 / d 10.1 | 70.88 | 70.84 |
| 4 | CH | 5.262 | d 10.1 / d 9.8 | 72.42 | 72.38 |
|  | CO | --- | --- | 171.88 | 171.87 |
|  | CH_3_ | 2.048 | s | 20.73 | 20.74 |
| 5 | CH | 4.099 | d 9.8 / q 6.3 | 68.07 | 68.03 |
| 6 | CH_3_ | 1.270 | d 6.3 | 17.74 | 17.74 |
| 1’ | C | --- | --- | 135.51 | 135.43 |
| 2’/6’ | CH | 7.638 | m | 129.52 | 130.04 |
| 3’/5’ | CH | 7.419 | m | 130.08 | 129.50 |
| 4’ | CH | 7.424 | m | 131.83 | 131.82 |
| 7’ | CH | 7.756 | d 16.0 | 147.78 | 147.74 |
| 8’ | CH | 6.673 | d 16.0 | 117.96 | 117.92 |
| 9’ | C | --- | --- | 167.37 | 162.32 |
| 1’’ | C | --- | --- | 135.42 | 135.35 |
| 2’’/6’’ | CH | 7.506 | m | 129.37 | 129.98 |
| 3’’/5’’ | CH | 7.340 | m | 130.00 | 129.35 |
| 4’’ | CH | 7.360 | m | 131.72 | 131.71 |
| 7’’ | CH | 7.618 | d 16.0 | 147.39 | 147.37 |
| 8’’ | CH | 6.422 | d 16.0 | 117.86 | 117.84 |
| 9’’ | C | --- | --- | 167.17 | 167.14 |

Figure 2: ^1^H-NMR spectrum of koelzioside in MeOH-d_4_; 201.1K

Figure 3: ^13^C-NMR spectrum of koelzioside in MeOH-d_4_; 201.1K

Table 2: ^1^H NMR and ^13^C NMR data of scrovalentinoside (**14**) in MeOH-d_4;_ 298,1 K

| **Position** | | **^1^H (ppm)** | **J_H.H_ (Hz)** | **^13^C (ppm)** | **^13^C (ppm)** (Giner et al., 1998) |
| --- | --- | --- | --- | --- | --- |
| 1 | CH | 5.099 | d 9.6 | 95.12 | 95.20 |
| 3 | CH | 6.398 | d 6.0 / d 1.8 | 142.49 | 142.40 |
| 4 | CH | 5.094 | d 6.0 / d 4.5 | 103.18 | 103.20 |
| 5 | CH | 2.488 | d 7.7 / d 7.9 / d 4.5 / d 1.8 | 37.17 | 37.10 |
| 6 | CH | 4.067 | d 8.2 / d 1.0 | 84.91 | 85.10 |
| 7 | CH | 3.671 | d 1.0 | 59.45 | 59.50 |
| 8 | C | --- | --- | 66.53 | 66.50 |
| 9 | CH | 2.589 | d 7.7 / d 9.6 | 43.29 | 43.30 |
| 10 | CH_2_ | 4.156 | d 13.1 | 61.40 | 61.60 |
|  |  | 3.820 | d 13.1 |  |  |
| β-Glc |  |  |  |  |  |
| 1 | CH | 4.777 | d 8.0 | 99.70 | 99.80 |
| 2 | CH | 3.265 | d 8.0 / d 9.3 | 74.82 | 74.80 |
| 3 | CH | 3.400 | d 9.3 / d 8.8 | 77.67 | 77.70 |
| 4 | CH | 3.255 | d 8.8 / d 9.9 | 71.78 | 71.70 |
| 5 | CH | 3.318 | d 9.9 / d 2.1 / d 6.7 | 78.65 | 78.50 |
| 6 | CH_2_ | 3.919 | d 2.1 / d 11.9 | 62.96 | 62.90 |
|  |  | 3.628 | d 6.7 / d 11.9 |  |  |
| α-Rha |  |  |  |  |  |
| 1 | CH | 5.076 | d 1.8 | 97.77 | 97.80 |
| 2 | CH | 5.304 | d 1.8 / d 3.4 | 71.23 | 71.40 |
|  | CO | --- | --- | 171.67 | 171.60 |
|  | CH_3_ | 2.163 | s | 20.69 | 20.80 |
| 3 | CH | 5.361 | d 3.4 / d 10.2 | 70.64 | 70.60 |
|  | CO | --- | --- | 171.64 | 171.60 |
|  | CH_3_ | 1.928 | s | 20.61 | 20.80 |
| 4 | CH | 5.170 | d 10.2 / d 9.9 | 71.99 | 72.10 |
| 5 | CH | 4.072 | d 9.9 / q 6.3 | 68.28 | 68.40 |
| 6 | CH_3_ | 1.217 | d 6.3 | 17.77 | 18.00 |
| 1’ | C | --- | --- | 128.10 | 127.90 |
| 2’/6’ | CH | 7.571 | d 8.8 | 131.24 | 131.20 |
| 3’/5’ | CH | 6.958 | d 8.8 | 115.48 | 115.50 |
| 4’ | C | --- | --- | 163.42 | 163.30 |
|  | OCH_3_ | 3.832 | s | 55.90 | 55.80 |
| 7’ | CH | 7.677 | d 15.9 | 147.39 | 147.40 |
| 8’ | CH | 6.375 | d 15.9 | 115.06 | 115.10 |
| 9’ | C | --- | --- | 167.93 | 167.90 |

Figure 4: ^1^H-NMR spectrum of scrovalentinoside in MeOH-d_4_; 201.1K

Figure 5: ^13^C-NMR spectrum of scrovalentinoside in MeOH-d_4_; 201.1K

Figure 6: HPLC of fraction A and hispidulin (mobile phase 2 and gradient 2, see experimental)


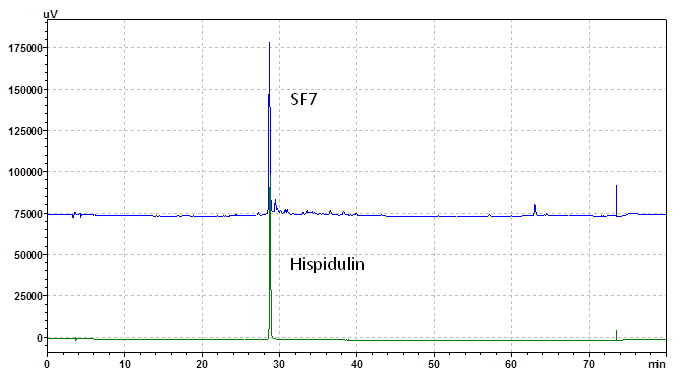


**Fraction A**

Figure 7: UV spectrum of the compound in fraction A eluting at 28.7 min. (left) and of hispidulin (28.8 min; right)


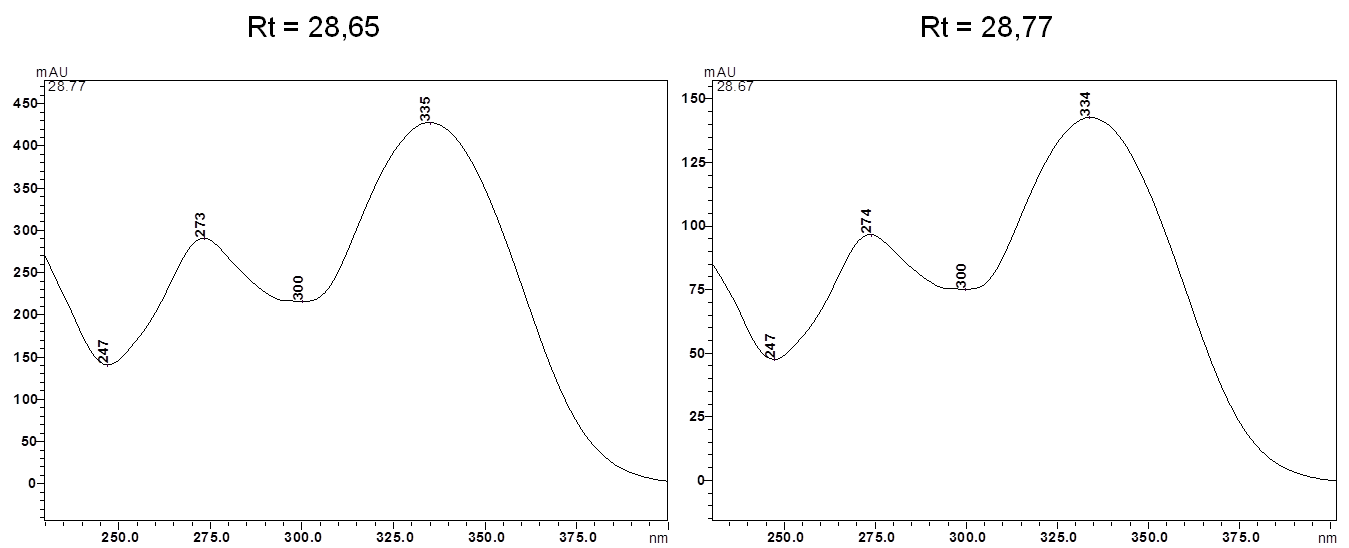

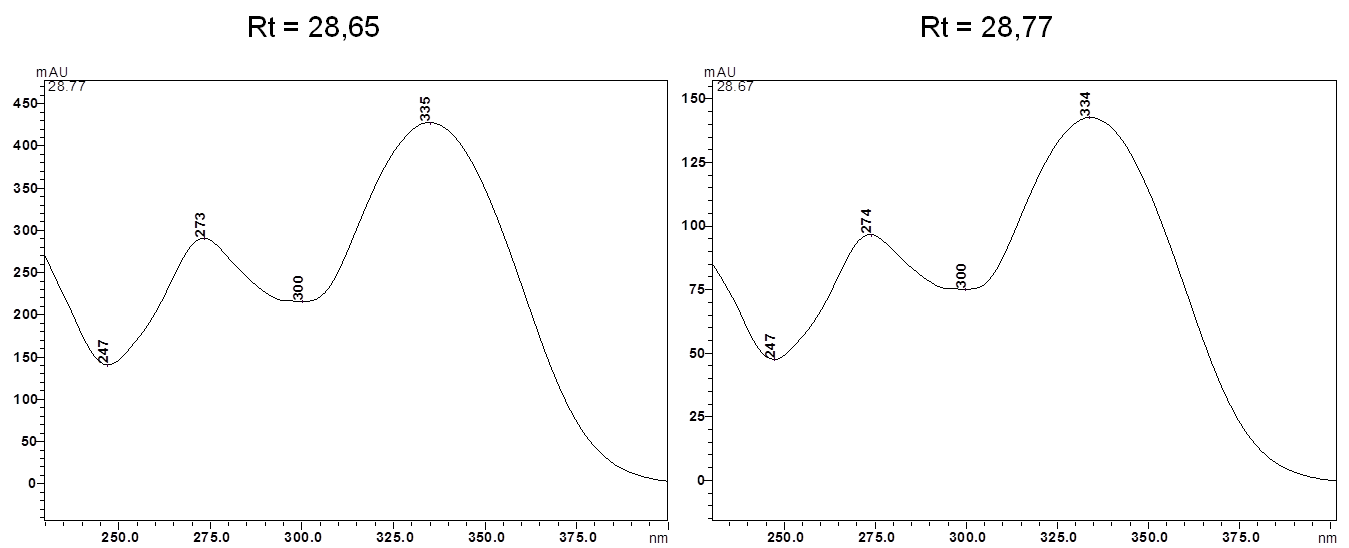


Figure 8: LC-MS^n^ data (positive ion mode, see experimental) of the compound coeluting with hispidulin in fraction A

Figure 9: HPLC of fraction g6 and verbascoside (mobile phase 2 and gradient 2, see experimental)

**Fraction g6**

**Verbascoside**

Figure 10: UV spectrum of the compound in fraction g6 eluting at 17.2 min. (left) and of verbascoside (17.2 min.; right)


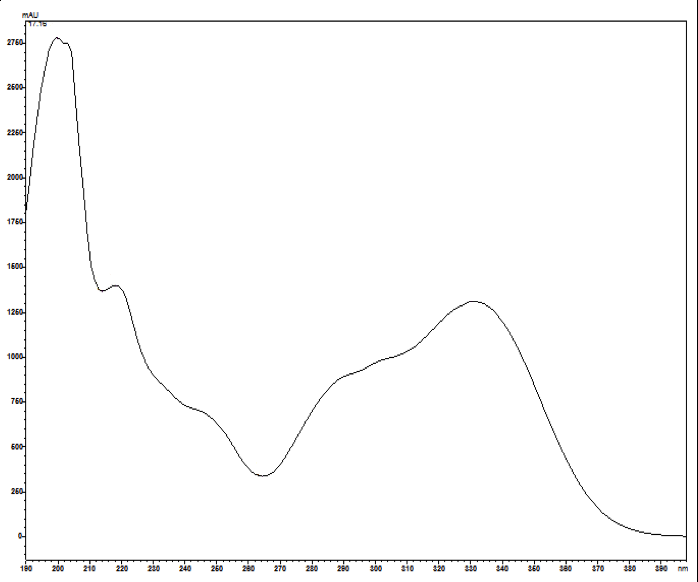

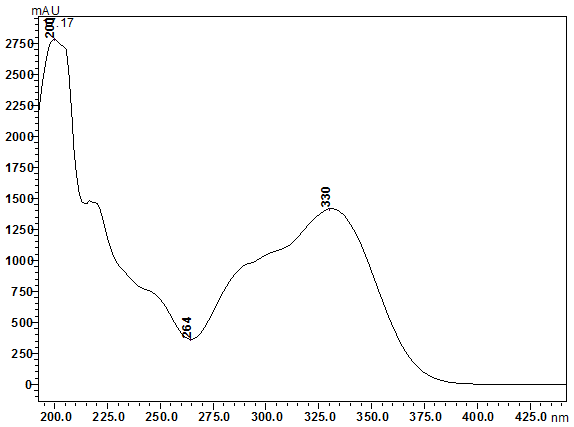


218

264

330

Figure 11: LC-MS^n^ data (negative ion mode, see experimental) of the compound coeluting with verbascoside in fraction g6

Figure 12: HPLC of fraction g10 and rutin (mobile phase 2 and gradient 2, see experimental)

**Fraction g10**

**Rutin**

Figure 13: UV spectrum of the compound in fraction g10 eluting at 18.3 min. (left) and of rutin (18.3 min.; right)

Figure 14: HPLC of fraction g10 and kaempferol-3-O-rutinoside (mobile phase 2 and gradient 2, see experimental)

**Fraction g10**

**Kaempferol-3-O-rutinoside**

Figure 15: UV spectrum of the compound in fraction g10 eluting at 19.2 min. (left) and of kaempferol-3-O-rutinoside (19.2 min.; right)

Figure 16: HPLC of fraction g19 and luteolin-7-O-glucoside (mobile phase 2 and gradient 2, see experimental)

**Fraction g19**

**Luteolin-7-O-glucoside**

Figure 17: UV spectrum of the compound in fraction g19 eluting at 20.1 min. (left) and of luteolin-7-O-glucoside (20.0 min.; right)

Figure 18: HPLC of a) fraction e22 and b) nepitrin (mobile phase 1 and gradient 1, see experimental)

a)

Nepitrin

b)

Nepitrin

Figure 19: UV spectrum of the compound in fraction e22 eluting at 37.2 min. (left) and of nepitrin (37.1 min., right)

Figure 20: LC-MS^n^ data (negative ion mode, see experimental) of the compound coeluting with nepitrin in fraction e22

Figure 21: LC-MS^n^ data (positive ion mode, see experimental) of the compound coeluting with nepitrin in fraction e22

Figure 22: ^1^H-NMR spectrum of homoplantaginin in DMSO-d_6_; 298K

Figure 23: ^13^C-NMR spectrum of homoplantaginin in DMSO-d_6_; 298K

Figure 24: ^1^H-NMR spectrum of 2”-O-acetyl-homoplantaginin in DMSO-d_6_; 298K

Figure 25: ^13^C-NMR spectrum of 2”-O-acetyl-homoplantaginin in DMSO-d_6_; 298K

Bhandari, S.P.S., Mishra, A., Roy, R. and Garg, H.S. (1992) Koelzioside, an iridoid diglycoside from *Scrophularia koelzii*. *Phytochemistry* 31, 689-691.

Giner, R.M., Villalba, M.L., Del Carmen Recio, M., Mainez, S., Gray, A.I. and Rios, J.L. (1998). A New Iridoid from *Scrophularia auriculata ssp. pseudoauriculata*. *J. Nat. Prod.* 61, 1162-1163.
